# Exercise-mobilized donor lymphocyte infusions enhanced with cytokine stimulation for the prevention and treatment of leukemic relapse after allogeneic hematopoietic cell transplantation

**DOI:** 10.3389/fimmu.2025.1563972

**Published:** 2025-06-12

**Authors:** London M. McDougal, Forrest L. Baker, Michael P. Gustafson, Emmanuel Katsanis, Richard J. Simpson

**Affiliations:** ^1^ School of Nutritional Sciences and Wellness, University of Arizona, Tucson, AZ, United States; ^2^ Department of Pediatrics, University of Arizona, Tucson, AZ, United States; ^3^ The University of Arizona Cancer Center, Tucson, AZ, United States; ^4^ Laboratory Medicine and Pathology, Mayo Clinic Arizona, Phoenix, AZ, United States; ^5^ Department of Immunobiology, University of Arizona, Tucson, AZ, United States; ^6^ Department of Medicine, University of Arizona, Tucson, AZ, United States; ^7^ Department of Pathology, University of Arizona, Tucson, AZ, United States

**Keywords:** hematological cancer, cell therapy, exercise immunology, exercise oncology, adrenergic receptors, cardiorespiratory fitness, NK-cells, cytokine-induced memory-like NK-cells

## Abstract

Donor lymphocyte infusions (DLI) are a standard therapy following allogeneic hematopoietic cell transplantation (alloHCT) for preventing and treating leukemic relapse in high-risk patients, particularly those with myeloid malignancies such as acute myeloid leukemia (AML), chronic myeloid leukemia (CML), and myelodysplastic syndrome (MDS). However, the efficacy of DLI remains suboptimal and is accompanied by a significant risk of life-threatening graft-versus-host disease (GvHD), highlighting the urgent need for strategies that enhance graft-versus-leukemia (GvL) effects while mitigating GvHD. We propose that engaging donors in an acute bout of exercise during peripheral blood lymphocyte collection represents a promising strategy to enhance GvL activity whilst mitigating the risk of GvHD. A single bout of cardiorespiratory exercise triggers catecholamine- and β_2_-adrenergic receptor-dependent mobilization of effector lymphocytes into the bloodstream, significantly increasing the proportion of GvL-promoting NK-cells and γδ T-cells relative to total CD3+ T-cells while reducing GvHD-promoting naïve CD4+ and CD8+ T-cells. Preclinical evidence suggests that these exercise-mobilized lymphocytes infiltrate tumors, exhibit enhanced leukemic control in xenogeneic mice, and display transcriptomic and proteomic profiles indicative of heightened anti-tumor immunity, migration potential and cytokine responsiveness. In this narrative review, we evaluate the advantages and limitations of DLI as a post-alloHCT therapy and propose the novel concept of exercise-enhanced donor lymphocyte infusions (DLI-X) as a simple and cost-effective strategy to augment GvL effects in preventing and treating leukemic relapse. Additionally, we propose that enriching DLI-X with NK-cell-enhancing cytokines (e.g., IL-12, IL-15, and IL-18) will create a novel therapeutic product, termed DLI-XS, with enhanced potency for post-alloHCT applications. We also discuss how DLI-X and DLI-XS, can be leveraged in combination with other post-transplant interventions to maximize GvL effects while minimizing GvHD risks. Finally, we explore the critical role of donor fitness (e.g. V̇O_2_max) in potentially influencing clinical outcomes of alloHCT and post-transplant cell therapies. This comprehensive integration of DLI-X and DLI-XS into existing treatment paradigms represents a promising avenue for enhancing therapeutic outcomes in leukemic relapse post-alloHCT and will underscore the transformative potential of exercise as an accessible and cost-effective adjuvant for DLI.

## 1 Introduction

Allogeneic hematopoietic cell transplantation (alloHCT) is a promising curative treatment for patients with hematologic malignancies. However, relapse remains a substantial and ongoing challenge limiting its long-term success for many patients. Donor lymphocyte infusions (DLI) represent a form of adoptive immune cell therapy administered to the recipient to mitigate or treat disease recurrence post alloHCT (1). DLI is used to amplify the graft versus leukemia (GvL) effect and has shown particular success in cases of chronic myeloid leukemia (CML) when addressing impending relapse, especially when detected at the cytogenetic (BCR-ABL) or molecular level (2–5). Unfortunately, the efficacy of DLI in achieving leukemic remission is limited, particularly in acute leukemias and lymphoid malignancies. Its application is further constrained by the risk of graft-versus-host disease (GvHD) - an often-serious complication where donor T-cells attack the recipient’s healthy tissues, resulting in significant morbidity and mortality (1). As such, there is an unmet need to enhance the GvL effects of DLI without increasing the risk of GvHD.

Compelling evidence underscores the pivotal role of physical activity in diminishing cancer risk and enhancing outcomes throughout cancer treatment (6–8). With each bout of exercise, there is an instantaneous and substantial mobilization and redistribution of effector lymphocytes between the blood and secondary lymphoid tissues. This process is intricately linked to the actions of hemodynamic shear stress and, more prominently, catecholamines signaling through β2-adrenergic receptors (ARs) (9). Lymphocytes exhibiting robust anti-tumor potential, including NK-cells, CD8+ T-cells, and gamma-delta (γδ) T-cells with phenotypes associated with migration and cytokine signaling, display elevated surface expression of β2-ARs. Consequently, these lymphocytes are preferentially mobilized during each bout of exercise, with the cumulative impact of repeated bouts of exercise (i.e. long-term training) manifests in heightened immune surveillance, fostering increased recognition and elimination of premalignant cells in the early stages of tumor development (7, 9). This phenomenon holds the potential to curtail tumor growth, a phenomenon observed in various murine tumor models and, more recently, in clinical trials involving cancer patients (10–13).

In this narrative review, we describe how a single bout of exercise alters the phenotype and function of blood lymphocytes and propose our assertion that harvesting lymphocytes from donor blood during the mobilization phase of acute exercise can yield a superior product for DLI, which we have termed ‘DLI-X’. The premise rests on the expectation that DLI-X will exert enhanced GvL effects without a corresponding rise in GvHD, therefore offering a promising advancement in cancer immunotherapy. In addition, we explore the potential synergies between DLI-X and *ex vivo* cell enhancement techniques, such as overnight cytokine stimulation, denoted herein as ‘DLI-XS’, as well as post-transplant therapeutics such as monoclonal antibodies that are designed to increase antibody-dependent cellular cytotoxicity (ADCC) of NK-cells. Finally, we discuss how the physical conditioning (e.g. V̇O_2_max) of the donor may impact the efficacy of DLI and alloHCT and how DLI-X and DLI-XS can be translated to the patient population.

## 2 Allogeneic hematopoietic cell transplantation

AlloHCT stands as the beacon of hope for many individuals with hematologic malignancies, with more than 9,000 alloHCT performed annually in the United States (14, 15). The procedure begins with chemotherapy preconditioning of the recipient and in some cases total body irradiation followed by infusion of hematopoietic stem cells from a healthy donor into the patient with a hematologic malignancy. These stem cells are sourced from bone marrow, umbilical cord blood or peripheral blood after mobilization of CD34+ hematopoietic stem cells using granulocyte colony-stimulating factor (G-CSF). Once infused, the donor stem cells migrate to the recipient’s bone marrow where they engraft and restore normal hematopoiesis.

The matching of human leukocyte antigens (HLA) molecules between the donor and the recipient is crucial for the success of alloHCT. HLA are proteins found on the surface of cells that play a key role in the immune system’s recognition of foreign substances. The genes that encode for HLA molecules are highly polymorphic and matching HLA between the donor and the recipient is crucial for the success of alloHCT. When HLA molecules on the donor cells closely match those of the recipient, the risk of graft rejection (the recipient’s immune system attacking the donor cells) and GvHD is reduced. HLA matching considers several key HLA loci, including HLA-A, HLA-B, HLA-C, HLA-DRB1, HLA-DPB1, and HLA-DQB1 (16, 17). A higher level of matching at these loci is associated with better transplant outcomes. Live donor sources considered in HCT may include: (i) Matched sibling donor (MSD); (ii) Matched unrelated donor (MUD); (iii) mismatched unrelated donor (MMUD) (usually limited to a single antigen) and (iv) Haploidentical (haplo) donor (one haplotype match and mismatched at the other haplotype). HLA matching is accomplished by considering HLA-A, B, C, DRB1 (8/8), HLA-A, B, C, DRB1, DQB1 (10/10) and 12/12 when DPB1 is also matched (18).

Siblings have a 25% chance of being an HLA match and 50% chance of being haploidentical, as each inherits one haplotype from each parent. When a MSD is unavailable, a MUD may be identified through national and international registries of volunteer donors. In cases where a fully matched donor cannot be found, transplantation may proceed with a MMUD or a haploidentical donor. Haploidentical transplants (haploHCT) typically using a parent, child, or sibling as the donor, have significantly improved donor availability for certain patient populations, particularly minorities. The use of post-transplant cyclophosphamide (PT-CY) as a GvHD mitigation strategy has facilitated successful transplantation despite HLA disparities (19–23). PT-CY plays a crucial role by eliminating both donor and recipient alloreactive T-cells, thus averting rejection and mitigating severe GvHD. However, while PT-CY effectively prevents GvHD, its immunosuppressive effects can inadvertently dampen the GvL response, leading to disease relapse, a significant factor contributing to treatment failure (23, 24). In this scenario, DLI or other adoptive cell therapies may be needed to enhance the GvL effect targeting residual leukemia in the recipient to achieve durable remission.

## 3 Donor lymphocyte infusions

Although alloHCT holds promise as a curative therapy, tackling relapse remains a formidable and enduring obstacle, and is responsible for the mortality of 37–57% of recipients (15, 25, 26). The presence of mixed chimerism, often seen after reduced intensity conditioning regimens, is characterized by the coexistence of donor and recipient hematopoietic cells. Mixed chimerism predisposes patients to relapse and/or graft rejection. DLI is often necessary to mitigate or treat disease recurrence and mixed chimerism following alloHCT (1, 27, 28). Alloreactivity resulting from HLA major or minor antigen mismatches or activating KIR mismatches contribute to the GvHD and GvL effects of DLI (29–31). Standard practice for DLI is to use freshly collected non-G-CSF mobilized and unmanipulated lymphocytes from the original stem cell donor. In certain cases, DLI may involve the use of lymphocytes that were cryopreserved following G-CSF mobilization of stem cells. Both approaches prioritize targeting leukemia through alloreactivity whilst balancing the risk of GvHD. Although G-CSF mobilization promotes a shift in T-cell polarization toward a more tolerogenic immune profile—potentially mitigating acute GvHD (32, 33), —it does not fully prevent it and has been associated with a higher incidence of chronic GvHD compared to unmanipulated DLI (34–39), likely reflecting distinct mechanisms underlying acute versus chronic disease pathogenesis.

Prophylactic DLI (proDLI) is a strategy that has gained traction and is increasingly being utilized post-alloHCT for high-risk hematologic malignancies (40–46). This involves infusing lymphocytes from the donor after alloHCT, strategically administered before the onset of relapse (e.g. ~90-days post alloHCT). Pre-emptive donor lymphocyte infusion (preDLI) is delineated by its administration to patients exhibiting either persistent minimal residual disease (MRD+) or the emergence of new MRD+. Conversely, therapeutic DLI (tDLI) is considered when given to patients experiencing overt relapse coupled with declining donor chimerism. Unfortunately, the efficacy of tDLI in achieving leukemic remission after alloHCT (tDLI) remains suboptimal, and its application is hindered by the potential risk of GvHD (47–49). Although data suggest that proDLI may reduce relapse and improve survival (50–52), direct head-to-head comparisons with tDLI are sparse, underscoring the need for prospective studies to guide optimal timing and application of DLI.

Chimeric antigen receptor (CAR) T-cells have largely replaced DLI for treating CD19+ leukemic relapse (e.g. B-ALL) after alloHCT, with high complete remission rates and durable responses reported in relapsed/refractory settings (53–57). However, CAR T-cell options for myeloid malignancies such as AML and CML remain limited, and DLI continues to be a central strategy for harnessing GvL effects in these diseases. Moreover, despite the notable success of CD19 directed CAR T-cells, they face inherent limitations, including dependence on autologous sources, challenges arising due to the emergence of CD19 escape variants, variable duration of persistence within the host, long manufacturing times, and the potential for severe complications like cytokine release syndrome (CRS) and immune effector cell-associated neurotoxicity syndrome (ICANS) (56, 57). Given these challenges, other immune effector cells—such as NK-cells, invariant natural killer T cells, γδ T cells, and macrophages—are increasingly being explored as alternative vehicles for CAR engineering and may eventually expand the repertoire of engineered cell therapies for cancer treatment (58). However, the use of these cells has additional limitations including suboptimal expansion, limited persistence, reduced cytotoxicity compared to αβ T cells, and the need for further optimization of manufacturing protocols and clinical efficacy (58). Additionally, the considerable costs associated with CAR-T therapies contribute to disparities among patients. DLI, characterized by its lower cost and lack of *ex vivo* manipulation requirements, is a practical and readily available option for a broader range of patients provided that the GvL effects can be increased without a corresponding risk in GvHD. This underscores the pressing need for innovative strategies aimed at enhancing the potency of DLI against both lymphoid and myeloid malignancies (59).

## 4 Exercise mobilizes immune cells with increased anti-tumor potential

Strong evidence underscores the central role of physical activity in reducing cancer risk and improving outcomes across various treatment modalities (6, 7). The benefits of exercise are multifaceted, spanning enhanced immune surveillance, reduced senescent T-cell populations, improved responsiveness to immunotherapy, better cardiovascular health, heightened insulin sensitivity, and improved quality of life (60). A widely accepted paradigm in exercise immuno-oncology is that the positive effects of exercise on anti-tumor immunity arise from the cumulative impact of each exercise bout (6, 7, 61). Each session of cardiorespiratory exercise—such as running, cycling, or rowing—induces an immediate and substantial mobilization and redistribution of effector lymphocytes between the blood and secondary lymphoid tissues. This process is driven by increases in hemodynamic shear stress and catecholamine signaling through β2-adrenergic receptors (β2-ARs) (9). Lymphocytes with potent anti-tumor properties, including NK-cells, CD8+ T-cells, and γδ T-cells, express high levels of β_2_-ARs, making them particularly responsive to exercise-induced mobilization (9). Our lab and others have shown that a single 20–30-minute bout of dynamic exercise at 50–80% of maximal oxygen uptake (V̇O_2_max) can mobilize total lymphocytes into circulation up to threefold, and lymphocyte subtypes such as NK-cells up to fivefold (62–66). Following exercise cessation, these cells rapidly exit the circulation to surveil peripheral tissues (67–69). When repeated consistently, this mobilization and redistribution process strengthens immune surveillance, potentially aiding in the detection and elimination of premalignant cells during early tumor development (6, 7, 9).

Murine cancer models provide robust evidence supporting this paradigm. In a landmark study, Pedersen et al. demonstrated that voluntary wheel running reduced tumor burden by 67% in a subcutaneous B16F10 melanoma model and dramatically reduced lung metastases when tumor cells were injected intravenously (10). Interestingly, these protective effects of exercise persisted in athymic mice lacking functional T cells; however, both *in vivo* NK-cell ablation and β1+β2-AR blockade using propranolol abolished these protective effects, underscoring the central role of NK cells and adrenergic signaling in mediating exercise benefits (10). In contrast, other studies highlight the critical role of CD8+ T cells in exercise-mediated tumor suppression. Rundqvist et al. showed that voluntary wheel running reduced tumor growth in the I3TC breast cancer model through increased frequencies of cytotoxic CD8+ T cells in tumors, spleens, and tumor-draining lymph nodes (11). Depleting CD8+ T cells eliminated these benefits, confirming their pivotal role. Similarly, Kurz et al. demonstrated that exercise-induced suppression of pancreatic ductal adenocarcinoma (PDAC) growth required CD8+ T cells (12). Depleting these cells reversed tumor suppression and abolished the associated increase in apoptotic tumor cells, indicating that CD8+ T cells are essential for the cytotoxic immune response triggered by exercise. Critically, it was found that exercise-induced increases in intra-tumoral CD8+ T cells rely on peripheral mobilization, driven by an epinephrine-mediated sympathetic spike, which activates lymphocyte mobilization. Adrenergic signaling was essential for this process, as propranolol eliminated the exercise-induced tumor protection and CD8+ T cell activation. The sphingosine-1-phosphate (S1P) and S1P-receptor (S1PR) gradient pathway, which regulates immune cell egress from blood and secondary lymphoid organs to sites of injury, was found to play a significant role in this mobilization. Inhibition of this pathway with Fingolimod prevented the exercise-induced expansion of CD8+ T cells both in the tumor and peripherally, abolishing exercise-mediated tumor protection, thereby highlighting the critical function of the S1P-S1PR gradient in the immune-modulatory effects of exercise in this murine model of pancreatic cancer (12).

Recent work from our group reinforces the critical involvement of β2-AR signaling and NK cells in exercise-induced tumor suppression. In a murine B-cell A20 lymphoma model, voluntary wheel running protected against tumor progression, an effect abolished by either β1+β2-AR blockade or NK-cell ablation (70). Blocking β1-AR alone, however, did not impact the anti-tumor effects, highlighting β2-AR signaling as the dominant adrenergic pathway. While NK cells were identified as key mediators of the exercise-induced tumor control, excised tumors from exercising animals exhibited a higher infiltration of CD8+ T cells and only a modest trend toward increased NK-cell infiltration compared to controls (70). This suggests that NK cells play a critical role in the early stages of the immune response, while CD8+ T cells may assume a more prominent role in controlling A20 lymphoma progression at later time points (71, 72).

Clinical evidence also supports the role of exercise in modulating anti-tumor immunity. Kurz et al. reported that patients with PDAC who completed a preoperative exercise regimen—including aerobic activity and strength training—exhibited significantly higher levels of tumor-infiltrating CD8+ T cells and a trend toward increased granzyme B (GZMB) expression compared to historical controls (12). Patients with high intratumoral CD8+ T cell or GZMB levels in the exercise cohort also demonstrated improved median overall survival, a benefit not observed in the control cohort. Similarly, Djurhuus et al. found that high-intensity interval training (HIIT) before surgery did not significantly increase tumor NK-cell infiltration in men with localized prostate cancer in the intention-to-treat analysis. However, a per-protocol analysis revealed significant increases in NK-cell infiltration, with a positive correlation between the number of exercise sessions and improvements in cardiorespiratory fitness, peak power output, and NK-cell infiltration (13). Taken together, these findings highlight the ability of exercise to mobilize and enhance NK-cell and CD8+ T-cell infiltration into both murine and human tumors, potentially amplifying their functional capacity and contributing to improved tumor control.

## 5 Exercise-enhanced donor lymphocyte infusion

Due to exercise-preferentially mobilizing lymphocytes (particularly NK-cells, CD8+ T-cells and γδ T-cells) with increased anti-tumor potential, our proposal hinges on the concept that extracting lymphocytes from donor blood during the mobilization phase of acute exercise can produce superior cellular candidates for DLI, which we refer to as ‘DLI-X’. This premise is based on the anticipation that DLI-X will exhibit heightened GvL effects without increasing risk of GvHD (6, 73), thus offering a promising advancement in the therapeutic options available to prevent and treat leukemic relapse after alloHCT. To evaluate the impact of DLI-X *in vivo*, we utilized an innovative transgenic immunocompromised non-obese diabetic (NOD)-scid IL2Rγnull (NSG) mouse model engineered with a human IL-15 knock-in gene to facilitate NK-cell persistence following adoptive transfer (62). Lymphocytes harvested from healthy human donors under both resting and exercise conditions were transferred into these mice, which were subsequently challenged with K562 leukemia cells to simulate the clinical scenario of employing DLI-X for preventing leukemic relapse post alloHCT (i.e. proDLI). Mice that received DLI-X demonstrated enhanced overall survival, a trend toward improved tumor-free survival, and a reduced tumor burden among surviving mice at 6 weeks when compared to those receiving standard DLI from the same donors. Notably, after the study, 48% of mice treated with DLI-X exhibited sustained survival with minimal tumor burden, compared to only 14% of mice treated with standard DLI (62). Crucially, our investigation revealed no discernible disparities in human immune cell engraftment, xenogeneic GvHD, or overall survival between mice administered DLI or DLI-X in the absence of K562 leukemia cells (62). This suggests that while DLI-X may enhance GvL effects, it does not appear to increase the risk of GvHD, at least in a xenogeneic setting. Although the precise mechanisms behind the improved GvL effects of DLI-X were not identified in this study, exercise-induced alterations in the immune cell composition of the cell collection, along with various molecular changes at the transcriptional and T-cell clonal levels are likely contributors and further outlined below.

### 5.1 Favorable immune cell composition in DLI-X

**Figure 1:**
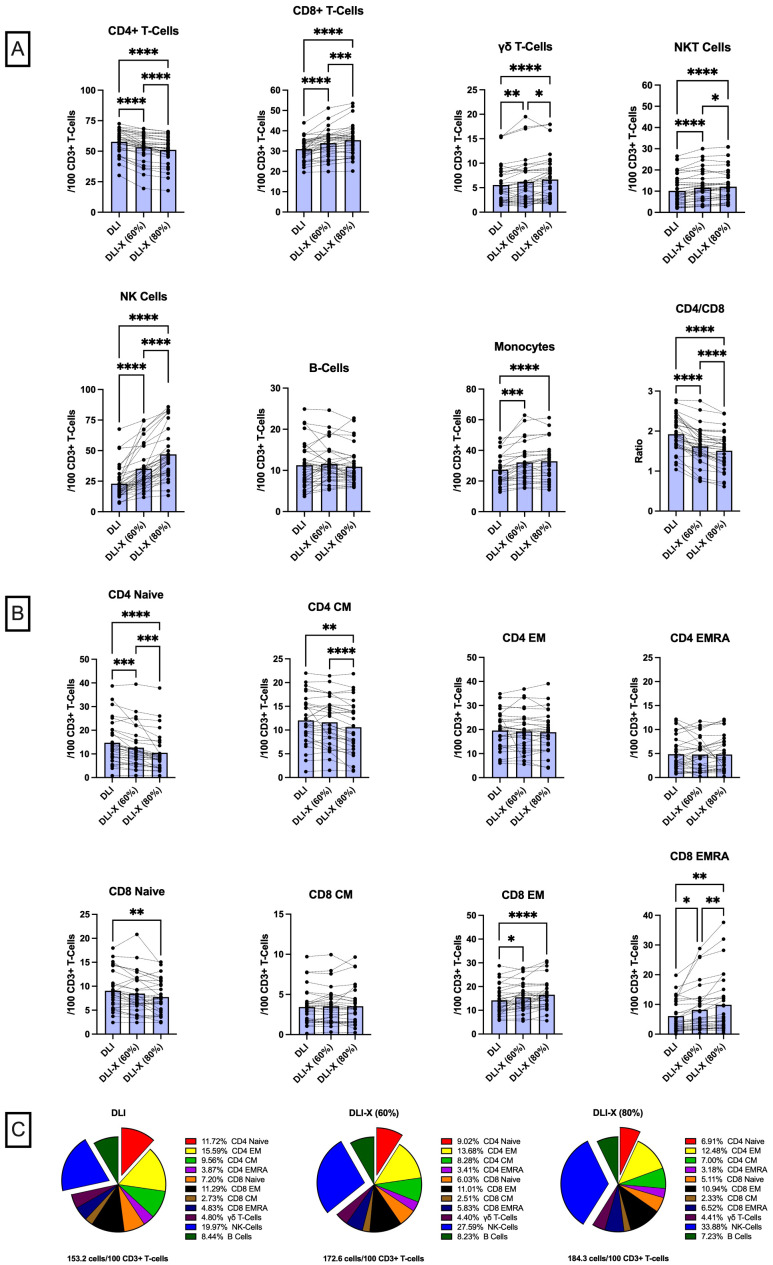
Immune cell composition of standard DLI and exercise-enhanced DLI-X at 60% and 80% VO2max, relative to total CD3+ T-cells in blood. Healthy participants (n=37) performed a 20-minute graded exercise test on a cycling ergometer, progressing from 50% to 80% V̇O_2_max. Blood samples collected at rest represented standard DLI, while those collected during exercise at 60% and 80% V̇O_2_max corresponded to DLI-X at moderate and vigorous intensities, respectively. Moderate and vigorous exercise increased the numbers (expressed as cells/100 CD3+ T-cells) of monocytes, NK-cells, CD8+ T-cells, γδ T-cells, and NKT-cells (CD3+/CD56+), while reducing CD4+ T-cells that led to a reduction in the CD4:CD8 T-cell ratio **(A)**. At 80% V̇O_2_max NK-cells, CD8+ T-cells, γδ T-cells and NK T-cells were further elevated, and CD4+ T-cell numbers and the CD4:CD8 T-cell ratio were further reduced compared to 60% V̇O_2_max. Vigorous exercise also reduced naïve CD4 and CD8 T-cells and central memory (CM) CD4 T-cells but increased effector memory (EM) and EMRA CD8 T-cells **(B)**. Overall, the total cell counts relative to 100 CD3+ T-cells increased by 19.4 and 31.1 cells in DLI-X at 60% and 80% V̇O_2_max, respectively **(C)**. This increase was primarily driven by higher NK-cell numbers alongside a reduction in CD4+ T-cells, particularly naïve CD4+ T-cells (exploded on the pie charts for illustration). The proportion of monocytes remained consistent (~18%) and is not shown on the pie charts. Data are from our ongoing clinical trial entitled “Exercise as an Immune Adjuvant for Allogeneic Cell Therapies (Allo-X)” (NCT06643221). Data were analyzed by one-way ANOVA with repeated measures and Tukey’s test for multiple comparisons and expressed as the mean with individual values (n=27–36 for each cell type). Significant difference indicated by * (p<0.05), ** (p<0.01), *** (p<0.001) and **** (p<0.0001).

The composition of lymphocyte subtypes within the DLI plays a critical role in determining patient outcomes (1). The main cells involved in GvL are effector lymphocytes such as NK-cells, CD8+ T-cells, and γδ T-cells (74–76); whereas the main cells involved in GvHD are naïve CD4+ and CD8+ T-cells, total CD4+ T-cells, B-cells and potentially central memory (CM) CD4+ T-cells (76–79). Several groups have therefore attempted to manipulate the immune cell composition of the DLI prior to infusion. CD3+ T-cell depletion of the lymphocyte collection can be used to effectively reduce GvHD, especially in HLA-mismatched HCT. Unfortunately, this delays immune reconstitution and places the patient at greater risk for infection. CD45RA+ T-cell depletion, a strategy designed to deplete mostly naïve T-cells, may provide improved immune recovery, viral protection, and decreased GvHD but is associated with reduced alloreactive GvL responses (80). Alpha-beta/CD19 depletion, a strategy designed to preserve γδ T-cells and NK-cells in peripheral blood alloHCT grafts, has been shown to reduce the risk of GvHD. However, this approach is associated with significant challenges, including delayed immune reconstitution, heightened susceptibility to infections, and an increased risk of graft failure (81, 82). These limitations have thus far hindered its widespread adoption as a method to enhance the safety and efficacy of DLI. An advantage of DLI-X over standard DLI is the natural enrichment of NK-cells, effector-memory (EM) CD8+ T-cells, and γδ T-cells (**Figure 1**). Concomitantly, there is a natural diminution of naïve CD4+ and CD8+ T-cells, CM CD4+ T-cells, as well as immunosuppressive T-regs that may dampen the GvL response. Because these changes in immune cell composition occur naturally *in vivo* during the exercise response, DLI-X may eliminate the need for *ex vivo* cell manipulation strategies that are not only labor intensive but may compromise efficacy (1). Moreover, retaining some yet smaller amounts of certain cell types (e.g. naïve CD4+ T-cells) may be advantageous for engraftment, persistence and the overall GvL effect (1).

To minimize the risk of GvHD, the number of lymphocytes in the DLI is normalized to the CD3+ T-cell count in the cell collection (55-75% of all lymphocytes) and the recipient’s weight. In this regard, the number of CD3+T -cells delivered is always controlled, but the numbers of T-cell subtypes (e.g. CD4+ and CD8+ αβ T-cells and γδ T-cells) and non-T-cell ‘bystander’ cells (e.g. NK-cells, B-cells, monocytes) received by the patient will vary based on their natural composition within the cell collection. Data from our Early Phase 1 trial “Exercise as an Immune Adjuvant for Allogeneic Cell Therapies (Allo-X)” (NCT06643221) involving healthy volunteers (**Table 1**) reveals that DLI-X could theoretically provide patients with an additional ~20-30 ‘bystander’ cells per 100 CD3+ T-cells compared to standard DLI if lymphocyte collections were obtained from donors during an acute bout of exercise at 60-80% V̇O_2_max (**Figure 1**). The enrichment of NK-cells by > 2-fold is considered significant, as NK cells are key contributors to anti-leukemia responses and are considered safe for allogeneic use due to their relative lack of GvHD induction (85–87). A recent study revealed a significant decrease in donor-derived NK cells among recipients receiving PT-CY, resulting in mature NK cells infused with unmanipulated grafts being lost upon PT-CY administration, blunting NK cell alloreactivity (88). These findings were supported by another recent study demonstrating preferential expansion of immature, functionally impaired NK cells and reduced mature NK-cell populations following haplo-HCT with PT-CY, further highlighting the disruption of NK-cell alloreactivity in this setting (89). Moreover, CD56+ NK cells with a target cell dose of at least 1 × 10^6^/kg given prophylactically following haploHCT with PT-CY resulted in absence of GvHD (90). There is also some evidence demonstrating NK-cells may protect against GvHD by killing host dendritic antigen-presenting cells (APCs) thus reducing donor T-cell activation against host tissues (52, 86). As such, the enrichment of NK-cells within the lymphocyte collection is one of the major advantages of DLI-X.

**Table 1:** Physical and demographic characteristics of the participants (n=37; 14 females) included from the ‘Allo-X’ trial (NCT06643221).

Variable	Mean	SD	Range
Age (Years)	30.5	5.7	19-43
Height (cm)	175.2	11.6	154.6-197.1
Mass (kg)	74.2	16.0	50.5-130.0
Body Mass Index (kg m^-2^)	24.0	3.3	18.3-36.0
Physical Activity Rating (PAR)^a^	4.86	1.38	2-7
Peak Cycling Power (W)	223.5	56.8	125-395
V̇O_2_max (ml kg^-1^ min-^1^)	40.7	9.0	18.4-53.7
V̇O_2_max Rating^b^	3.53	1.54	1-6

a PAR on a scale from 1 (None) to 7 (vigorous) was determined in accordance with the procedures described by Jackson et al. (83).

b V̇O_2_max rating determined by assigning a numerical core of 1 (‘very poor’) to 6 (‘superior’) in accordance with ACSM age and sex-adjusted score categories of cardiorespiratory fitness (84).

Within the T-cell compartment, DLI-X has a ~21% lower CD4:CD8 ratio compared to DLI (**Figure 1**). This is considered advantageous, as a higher CD4:CD8 T-cell ratio in donor lymphocyte collections is generally predictive of a higher risk of GvHD, especially in the mismatched/haploidentical setting (91, 92). Moreover, the numbers of naïve cells within both CD4+ and CD8+ T-cells was decreased in DLI-X by up to ~28.6% and ~15.4%, respectively (**Figure 1**). This was coupled with a shift toward an increase in memory CD8+ T-cells but not CD4+ T-cells within DLI-X. Additionally, CM CD4+ T-cells decreased by up to ~13.2% in DLI-X. Studies in mice have demonstrated that naïve CD4+ T-cells alone are capable of causing GvHD (93), while mice receiving memory CD8+ T-cells can eradicate malignant cells without inducing GvHD (94). In a clinical trial involving 15 patients with disease relapse after alloHCT, participants received a manipulated DLI product comprising CD45RA-depleted and CD8+ memory T-cell-enriched infusions at escalating doses (1×10^6^ to 10×10^6^ cells/kg). The infusion resulted in a low incidence of GvHD and a 67% response rate, including seven complete responses, with a median overall survival of 19.6 months (95). Taken together, this indicates that the increased composition of memory CD8+ T-cells in DLI-X would be advantageous by offering enhanced GvL effects and potentially contributing to a lower incidence of GvHD. While the enrichment of CD8+ T-cells in DLI-X was driven by cells exhibiting an EM or a CD45RA+ EM (EMRA) phenotype (up to ~16.9% and ~62.3% compared to DLI, respectively), we have shown previously that cells with this phenotype are preferentially mobilized to the blood during exercise by a larger magnitude in individuals with persistent viruses such as CMV, and that a portion of the mobilized cells are specific to CMV and other viral antigens (96–98). Given that post-transplant viral infections remain a significant cause or morbidity and mortality after alloHCT (99), DLI-X may provide additional CD8+ T-cell mediated viral protection over standard DLI.

DLI-X also has higher numbers of γδ-T-cells, with increases up to 21.8% compared to DLI (**Figure 1**). γδ-T-cells represent a specialized group of T-cells that contribute to the activation of inflammatory responses within both the innate and adaptive immune system (100). Like NK cells, γδ-T-cells do not promote GvHD but provide a strong GvL effect and show promise as a therapeutic cell product in preclinical studies and early phase clinical trials of hematological malignancies (101, 102). Importantly γδ T-cells can function as antigen-presenting cells by internalizing and processing antigens, expressing MHC class II molecules, and stimulating the proliferation and activation of NK-cells and CD8+ T cells (103, 104). Finally, we have shown previously that exercise does not mobilize CD4+/CD25high/CD127dim T-regs (62), thus having a reduced frequency in DLI-X. Although T-regs play an important role in suppressing the alloreactive GvHD response, they can also inhibit GvL effects and several trials have aimed to enhance GvL responses by depleting CD25+ T-regs before administering tDLI, resulting in improved overall response rates to disease relapse.

In our ‘Allo-X’ trial, we examined the effects of both moderate (60% V̇O_2_max) and vigorous (80% V̇O_2_max) exercise on altering the immune cell composition of DLI-X (**Figure 1**). Crucially for the allogeneic donor population—who are likely to have a wide range of cardiorespiratory fitness levels—all cell composition shifts occurred during moderate-intensity exercise, though generally of lesser magnitude compared to those observed during vigorous-intensity exercise. This suggests that clinically meaningful changes in immune cell composition can be attained even with moderate-intensity exercise. Notably, all participants tested to date (n = 37) were able to complete the graded protocol up to and including sustained efforts at 80% V̇O_2_max, regardless of their fitness level—which ranged from ‘poor’ to ‘superior’—and their self-reported physical activity score, which ranged from 2 (infrequent light activity) to 7 (high-intensity exercise ≥5 days/week), as assessed using the Jackson Physical Activity Rating Scale (**Table 1**). This indicates that the protocol is not only effective but also feasible across diverse donor profiles. However, we observed substantial inter-individual variability in the magnitude of immune cell mobilization—particularly at higher intensities—which may be influenced by baseline fitness, catecholamine responses, β_2_-adrenergic receptor sensitivity, sex, age, and habitual training status. Future analyses from the complete Allo-X cohort (n > 200) will be instrumental in identifying these underlying physiological and biological predictors of mobilization responsiveness, which will help refine exercise prescriptions for diverse donor populations.

### 5.2 Phenotypic and molecular changes within immune cell subtypes in DLI-X

In addition to immune cell composition shifts within DLI-X, phenotypic and transcriptomic changes are evident in subsets of both CD8+ T-cells, γδ T-cells, and NK-cells suggesting a potential role in enhancing GvL effects at the individual cell level (62, 105). We performed the first transcriptomic analysis of exercise-mobilized lymphocytes at single-cell resolution and found that mobilized EM CD8+ T-cells, NK-cells, and γδ T-cells, exhibit differentially expressed genes and enriched gene sets associated with anti-tumor activity (62). These include functions such as cytotoxicity (e.g. PRF1, NKG7, GZMA, GZMB, GZMH), migration/chemotaxis (e.g. CX3CR1, CLIC1), activation (e.g. PTPRCAP), catecholamine G protein-coupled receptor signaling (e.g. SLC9A3R1, PLEK), antigen presentation/processing and, crucially for DLI, alloreactivity, exemplified by an enrichment of genes involved in GvHD/GvL responses. It is not conclusively known, however, if these represent intrinsic changes at the individual cell level, or if they reflect inherent differences between resting and exercise-mobilized cells with overlapping phenotypes. Collectively, these findings indicate that favorable shifts in cell composition and possibly phenotypic or functional changes at the single-cell level contribute synergistically to enhancing the potency of DLI-X.

Abundant preclinical evidence supports the scientific rationale that DLI-X holds promise for augmenting GvL effects when employed as a post alloHCT therapeutic. Over the past decades, numerous studies have demonstrated heightened cancer cell killing *in vitro* when lymphocytes collected during acute exercise are utilized as effector cells. For instance, our research revealed that lymphocytes obtained during a vigorous 30-minute exercise session exhibited a 1.3 to 2.5-fold increase in killing efficacy against various allogeneic hematological cancer cell lines compared to lymphocytes collected under resting conditions from the same donors (63, 106). Notably, this enhanced effect was predominantly attributed to the increased presence of NK cells among the mixed lymphocyte population, as evidenced by the elimination of exercise-induced benefits when adjusting for the number of dead cancer cells relative to the number of NK cells. It is important to acknowledge, however, that this study did not use purified NK-cells, which may have demonstrated enhanced cytotoxicity within the NK-cell compartment independently of increased NK-cell numbers (63, 106). Nevertheless, this observation does not diminish the clinical potential of DLI-X, as a higher number of NK cells relative to T cells in PBHC collections is indicative of more favorable outcomes following alloHCT (107, 108). Furthermore, our investigation revealed strong correlations between exercise-induced alterations in target cell killing of multiple myeloma (U266) and lymphoma (221.AEH) cells and phenotypic shifts within the NK cell compartment, particularly changes in the expression of the activating receptor NKG2C and the inhibitory receptor NKG2A (63, 106). This suggests that the proportional changes in specific subtypes within the NK-cell compartment, which exhibit enhanced cytolytic activity, may also play a role in the heightened cytotoxic effects observed with DLI-X.

The diversity of the T-cell receptor repertoire may also influence clinical outcomes of DLI. In a small trial involving 19 patients who received DLI post-alloHCT, a GvL response (remission or stable disease) was observed in 14 patients, eight of whom developed acute GvHD of grade III or higher (109). When assessing the GvL response, there was a trend indicating that remission induction was associated with a decrease in the CD8+ T-cell repertoire by day 28 compared to pre-DLI levels. Specifically, responsive patients exhibited a reduction in CD8+ T-cell diversity of approximately 28%, compared to less than 1% in those showing no GvL effect (109). We recently showed that a single exercise session significantly reduces the diversity of the T-cell repertoire in blood, with exercise preferentially mobilizing the most dominant clones in the repertoire (96). While it remains to be fully established whether the donor T-cell repertoire can predict GvL and GvHD responses in patients after DLI, these exercise-induced alterations in the T-cell repertoire may offer therapeutic advantages for DLI-X, potentially enhancing CD8 T-cell mediated GvL responses.

## 6 Enhancing NK-cell and γδ T-cell enrichment in DLI-X through augmented β2 adrenergic receptor signaling

The enrichment of NK-cells within DLI-X is primarily influenced by the actions of catecholamines, particularly epinephrine and the neurotransmitter norepinephrine, which activate β2 adrenergic receptors (β2-AR) that tend to be overexpressed on cytotoxic lymphocytes, including NK-cells, CD8+ T-cells, and γδ T-cells (9). Within these lymphocyte subsets, β2-AR expression is particularly heightened in phenotypes associated with differentiation and cytotoxicity. Additionally, organs like the spleen, which are significant contributors to the mobilization of leukocytes into the bloodstream during exercise, are regulated by adrenergic signals (9). In a randomized double-blind placebo-controlled crossover trial, we administered either a non-selective β1+β2-AR antagonist (nadolol) or a selective β1-AR antagonist (bisoprolol) to healthy participants three hours before a 30-minute bout of cycling exercise at +10% of the blood lactate threshold (64). The mobilization of various immune cell types, including total NK-cells, CD57+ NK-cells, CM, EM and EMRA CD8+ T-cells, as well as non-classical monocytes and γδ T-cells, was significantly reduced or abolished under nadolol compared to both bisoprolol and placebo. Importantly, cardiovascular responses such as heart rate and blood pressure that may also contribute to leukocyte mobilization through increased hemodynamic shear stress (110), were not different between the bisoprolol and nadolol trials (64). This suggests that β2-AR signaling predominantly governs the exercise-induced mobilization of these cell types into the bloodstream.

The mechanisms for this are largely due to the β2-AR induced disruption of lymphocyte adhesion to the endothelium. Activation of β2-AR leads to increased levels of cyclic adenosine monophosphate (cAMP) within the cell through the activation of adenylyl cyclase (AC). Elevated cAMP levels then activate protein kinase A (PKA), which phosphorylates various intracellular targets, including proteins involved in leukocyte adhesion (9). One of the key effects of PKA activation is the inhibition of integrin activation and affinity, particularly LFA-1 (lymphocyte function-associated antigen-1) and Mac-1 (macrophage-1 antigen), which are essential for leukocyte adhesion and transmigration across endothelial barriers. Epinephrine-mediated activation of β2-AR can also lead to decreased expression of adhesion molecules on endothelial cells, such as ICAM-1 (intercellular adhesion molecule-1) and VCAM-1 (vascular cell adhesion molecule-1), which are ligands for leukocyte integrins (111). This reduction in adhesion molecule expression contributes to decreased leukocyte adhesion to endothelial cells, facilitating their mobilization to the bloodstream. Epinephrine also induces vasodilation through activation of β2-AR on vascular smooth muscle cells. This vasodilatory effect can reduce the contact between leukocytes and endothelial cells, thus decreasing the opportunity for leukocyte adhesion (111).

In a subsequent study, we found that bisoprolol enhanced the mobilization of NK-cells compared to placebo across multiple exercise intensities ranging from 50-80% V̇O_2_max (70). Even at light-to-moderate exercise intensities (e.g., 50-60% V̇O_2_max), the number of NK-cells recruited to the bloodstream with bisoprolol resembled the levels observed during vigorous exercise (e.g. ~70% V̇O_2_max) with a placebo. Moreover, bisoprolol preferentially increased the mobilization of NKG2D+/NKG2A- NK-cells, indicating that this amplification of NK-cell mobilization was driven by the more cytotoxic subtypes. Importantly from the perspective of DLI-X, bisoprolol did not increase T-cell mobilization and thereby evokes higher NK/T-cell ratios. This augmented NK-cell mobilization is likely attributed to bisoprolol’s blockade of β1-AR, which increases epinephrine availability for β2-AR signaling, thereby facilitating NK-cell recruitment during exercise.

There are two major downstream signaling pathways following β2-AR signaling; the canonical cAMP-PKA pathway, which, phosphorylates various downstream targets, including ion channels, enzymes, and transcription factors, leading to a wide range of cellular responses, and the β-arrestin pathway which initiates signaling cascades independent of G-protein activation (9). The β1+β2-AR antagonist nadolol is known to inhibit downstream signaling via both pathways, whereas carvedilol (also a β1+β2-AR antagonist) inhibits cAMP-PKA but not β-arrestin signaling. We found that nadolol and carvedilol were equally effective in blocking the mobilization of effector lymphocytes to blood during exercise, indicating that downstream cAMP-PKA signaling is indeed the major pathway involved (70). We then attempted to further mobilize NK-cells during exercise by amplifying lymphocyte cAMP-PKA signaling using the phosphodiesterase-4 (PDE-4) inhibitor, roflumilast. PDE-4 is responsible for degrading nucleotides such as cAMP and cyclic guanosine monophosphate (cGMP) following β2-AR activation (112). By blocking PDE-4 in lymphocytes, roflumilast increases cAMP signaling by preventing cAMP hydrolysis, leading to higher intracellular levels of cAMP and subsequent activation of PKA-mediated signaling pathways (112). While combining roflumilast with bisoprolol did not increase NK-cell mobilization during exercise beyond what was already augmented with bisoprolol, we did find that this combination further increased the mobilization of CD8+ T-cells and γδ T-cells at exercise intensities equal to or greater than 60% V̇O_2_max (70). These findings suggest that selective β1-AR antagonists and PDE4 inhibitors could be administered within a few hours prior to exercise to augment β2-AR signaling and further enrich NK-cells and γδ T-cells, whilst concomitantly reducing the CD4/CD8 ratio within DLI-X, all of which are associated with better outcomes after alloHCT.

Administering bisoprolol and roflumilast could prove beneficial, especially for donors with low exercise tolerance, who may struggle to perform vigorous exercise for a sufficient duration to collect mobilized lymphocytes for transplantation (9). It may also be possible to use a synthetic β2-AR agonist in leu of exercise to enrich DLI products with NK-cells. The non-selective β1+β2-AR agonist isoproterenol (ISO) has been shown to preferentially mobilize cytotoxic lymphocytes to blood akin to exercise (105, 113). Indeed, we found that ISO infusion at 50ng/kg/min evoked a preferential mobilization of CD56dim NK-cells with an activated phenotype (e.g. NKG2D+/NKG2A-) akin to acute exercise at 70% V̇O_2_max (70). Moreover, allogeneic γδ T-cells expanded after ISO infusion showed enhanced anti-tumor activity *in vitro* and *in vivo* against the CML target cell line K562 when compared to γδ T-cells expanded from resting blood cells (105). We showed recently that ISO infusion can evoke major changes in peripheral blood stem cell collections after G-CSF mobilization, increasing the number of CD34+ HSCs and the NK-T-cell ratio, while simultaneously reducing the number of B-cells and naïve CD4+ and CD8+ T-cells (114). We also found that the G-CSF+ISO mobilized cells were superior at killing leukemia targets *in vitro* and controlling human leukemic burden in NSG-IL15 mice. Importantly, mice receiving G-CSF+ISO mobilized cells had improved survival and less xenogeneic GvHD compared to mice receiving standard G-CSF mobilized cells from the same donors (114).

## 7 Further enhancing the potency of DLI-X with NK-cell targeted cytokines

NK-cells stand out as prime candidates for allogeneic cell therapy due to their ability to identify and eliminate cancer cells without causing GvHD (115, 116). Furthermore, they can be incorporated into combination therapies designed to exploit their antibody-dependent cellular cytotoxicity (ADCC) functions. Mature/cytotoxic NK-cells, characterized by their CD56dim/CD16+ phenotype, exert direct cytotoxic effects against tumors via ADCC, which is facilitated by the binding of CD16 to the Fc region of IgG1 antibodies (115, 116). Moreover, NK-cells express a variety of activating and inhibitory receptors, and their activation hinges on the delicate balance of signals received through these receptors. The “missing-self” hypothesis suggests that NK-cell activation is favored in instances where there is a loss or downregulation of MHC-I molecules on tumor cells, thereby decreasing inhibitory signals. This recognition is facilitated by killer-cell immunoglobulin-like receptors (KIR), which detect self HLA molecules expressed by healthy cells (115, 116). NK-cells are promptly activated upon encountering cells with a diminished expression of HLA (e.g. tumor cells) or, in the case of allogeneic cell therapy, when the HLAs on the host tumor do not match those of the donor. Indeed, KIR mismatching has been demonstrated to enhance NK-cell mediated GvL effects, resulting in more potent NK-cell therapies against various hematological malignancies, including AML, CML and MM (115). Conversely, “stress-induced” NK-cell activation occurs when stress ligands such as MIC-A/B or PVR are upregulated on tumor cells, triggering activating receptors such as CD94/NKG2D or DNAM-1 (116). However, tumors can avoid detection and destruction by NK-cells by binding to their immune checkpoints, like NKG2A, TIM-3, TIGIT and CD96 (116). Despite their potential, allogeneic NK-cell therapy has its drawbacks, including the necessity for *in vitro* expansion to attain adequate numbers for transfer, a process that can take several weeks and may lead to exhaustion (116). Additionally, allogeneic NK-cells have a short half-life and often fail to persist *in vivo* after adoptive transfer (115). While IL-2 administration has been utilized to support NK-cell persistence post-transfer, its use can elicit various side effects and expand regulatory T-cells, which dampen the anti-tumor immune response (115, 116).

### 7.1 Cytokine-induced memory-like NK-cells

Attempts have been made to enhance the efficacy of NK-cells in DLI products without resorting to *ex vivo* expansion or genetic engineering. Extensive research has explored the impact of short-term (e.g. 12-16h) *ex vivo* cytokine priming on the anti-leukemic potential of NK-cells, revealing how specific combinations of cytokines can synergize to generate more potent cell products (117). Notably, the blend of IL-12, IL-15, and IL-18 has demonstrated the ability to generate memory-like properties in NK-cells (118, 119). These properties entail heightened IFN-γ production upon subsequent restimulation with cytokines or tumor cells. Referred to as cytokine-induced memory-like (CIML) NK-cells, they exhibit antigen-independent behavior, heightened reactivity, and distinct phenotypic alterations. Specifically, CIML NK-cells have an increased expression of the α chain of the IL-2 receptor, allowing them to proliferate more vigorously in response to IL-2 stimulation. Nutrient transporters including the transferrin receptor CD71 are upregulated on CIML NK-cells as they increase their metabolic activity following cytokine priming (118, 119). Although CD16 expression is decreased on CIML NK-cells, they still maintain capacity for ADCC and CD16 expression is usually restored within a few days of cytokine stimulation. Similarly, KIRs (isoforms of CD158) are down regulated after cytokine priming but restored within a few days following IL-2 stimulation. This indicates that CIML NK-cells undergo transient phenotypic and metabolic changes during their priming phase, which may give rise to the acquisition of long-term memory-like properties (118, 119). Moreover, CIML NK-cells appear to be more resistant to the immunosuppressive effects of TGF-β, which is secreted by tumors to impair cytotoxic lymphocytes through the TGF-β receptor and downstream SMAD signaling. TGF-β has been shown to downregulate important NK-cell activating receptors (e.g. DNAM-1, NKG2D, NKp30), chemokine receptors (e.g. CXCR3, CXCR4 and CX3CR1) and the synthesis of granzyme b, perforin and IFN-γ to impair NK-cell migration and cytotoxicity (118, 119). Moreover, CIML NK-cells have reduced TGF-β receptor and SMAD protein expression thus making them more resistant to the suppressive effects of TGF-β and thereby allowing them to have enhanced potency (118, 119).

The use of CIML NK-cells as an adoptive cell therapy is attractive because of their desirable characteristics, including manufacturing simplicity (overnight stimulation of purified NK-cells with IL-12/15/18), longer lifespan, enhanced cytotoxicity, and lack of GvHD (120). A preclinical study in murine A20 lymphoma showed increased survival and delayed tumor progression in mice that received CIML NK-cells compared to mice receiving control NK-cells and was attributed to their sustained activation/cytotoxicity and proliferative potential (121). Translational studies involving human CIML NK-cells were found to have enhanced IFN-γ production and cytotoxicity against K562 leukemia cell lines or primary human AML blasts *in vitro* (122). Moreover, CIML NK-cells were found to exert better control of K562 leukemia growth in NSG mice, which resulted in prolonged survival (123). The same group performed a first in-human Phase I clinical trial, demonstrating that adoptively transferred CIML NK-cells proliferated and persisted and demonstrated GvL effects in patients with relapsed/refractory AML, resulting in clinical responses in five of nine evaluable patients including four who had achieved complete remission (123). A follow up report of this trial with 15 evaluable patients revealed that CIML NK-cell therapy resulted in an overall response rate of 67% with 47% of patients achieving complete remission, without causing cytokine release syndrome, GvHD or neurotoxicity (124). Unlike other NK-cell products that typically exhibit poor persistence following adoptive transfer, infusion of CIML NK cells has been shown to induce a rapid 10- to 50-fold *in vivo* expansion, with persistence sustained over several months (120).

Combining CIML NK-cells with DLI has been considered as a synergistic therapy to treat leukemic relapse after alloHCT. In a recent clinical trial conducted at Washington University (NCT03068819), the efficacy of standard DLI supplemented with CIML NK-cells was investigated as a salvage therapy for relapsed AML patients post-alloHCT (125). The integration of these two treatment modalities, sourced from the same donors, was anticipated to enhance the GvL effect and prolong the persistence of NK-cells by circumventing rejection by T-cells. Nine pediatric and young adult patients received a treatment regimen consisting of fludarabine, cytarabine, and filgrastim. Two weeks later, they received DLI, followed by CIML-NK-cell administration the next day. Initial findings demonstrated that 4 out of 8 evaluable patients achieved complete remission by day 28. Notably, two patients sustained a durable remission for over 3 months, with one individual remaining in remission for more than 2 years, with no significant toxicities reported. These outcomes were accompanied by 10-50-fold CIML-NK-cell expansion *in vivo*, and their presence and functional responses were maintained >3 months. These findings indicate that the combined approach of DLI and CIML NK-cells may yield superior results compared to either modality alone, although this remains to be confirmed through a randomized control trial.

It is plausible that combining DLI-X with CIML NK-cells from the same collection could yield more potent anti-leukemic effects, whether used prophylactically or therapeutically. However, it remains uncertain whether exercise-mobilized NK-cells can be harnessed to generate superior CIML NK-cells. Nonetheless, our observations of transcriptomic and phenotypic changes within DLI-X NK-cells, indicative of heightened alloreactivity, cytotoxicity, and cytokine responsiveness (62), suggest that NK-cells from DLI-X may demonstrate increased efficacy following overnight triple cytokine stimulation, even on a per-cell basis. Additionally, some researchers have explored depleting GvHD causing αβ T-cells and B-cells from cell collections before triple cytokine stimulation (126). By preserving γδ T-cells and NK-cells, it is hypothesized that these two cell types will synergize to achieve superior results while minimizing the risk of GvHD (104, 127). Indeed, our recent work has demonstrated that γδ T-cells expanded from exercise-mobilized lymphocytes exhibit enhanced effector phenotypes and cytotoxic properties against various hematological tumors *in vitro* and *in vivo* when using xenogeneic mouse models of human CML (105, 128). Specifically, exercise-expanded γδ T-cells showed increased surface expression of several activating receptors such as NKG2D, TRAIL, and DNAM-1, with the latter found to mechanistically contribute to the enhanced cytotoxic effects against K562 leukemia through its ligation with PVR and Nectin-2 (105). Intriguingly, the effects of exercise on γδ T-cell mobilization and expansion were inhibited with β1+β2-AR blockade but not β1-AR blockade alone, and the effects of exercise on γδ T-cell expansion, phenotype, and cytotoxicity were replicated with ISO infusion (105). These findings provide compelling evidence that catecholamine-mediated adrenergic signaling plays a pivotal role in the adjuvant effects of exercise in manufacturing potent cell products for immunotherapy. This work also indicates that γδ T-cells, given their exercise responsiveness, could be playing an important role in the enhanced GvL effects of DLI-X.

### 7.2 Cytokine-stimulated exercise-enhanced donor lymphocyte infusions

A promising alternative to administering DLI followed by CIML NK-cells the next day (125) is to cultivate CIML NK-cells within DLI-X, leveraging the natural enrichment of NK-cells within the cell collection. This approach could streamline the generation process by eliminating the necessity for NK-cell purification, capitalizing on the presence of γδ T-cells in the cell collection, minimizing the number of adoptive transfers from two to one, and fostering enhanced synergy with cytokine-primed T-cells. We posit that stimulating DLI-X with cytokines *ex vivo* prior to adoptive transfer, referred to herein as DLI-XS, will provide a more robust post-transplant therapy against leukemia compared to both CIML NK-cells and standard DLI supplemented with CIML NK-cells.

To optimize the generation of a potent DLI-XS product for eliciting GvL effects, identifying the most effective cytokine stimulation cocktail is paramount. Initially, using the triple cytokines IL-12/15/18, known for activating CIML NK-cells, is recommended. However, augmenting this with additional cytokines such as IFN-α or IL-21 could potentially enhance the priming of NK-cells, γδ T-cells, and CD8+ T-cells within DLI-X (126, 129, 130). For example, short-term stimulation of blood lymphocytes with IFN-α ex vivo has been shown to enhance NK-cell degranulation and cytotoxicity against K562 leukemia cells and increase MIP-1β and IFN-γ expression (131). Incorporating IFN-α into the cytokine-priming cocktail for DLI-XS may also assist in preventing GvHD by inhibiting IL-2 and IL-7 mediated CD4+ T-cell proliferation (132). Importantly, IFN-α could also be delivered *in vivo* following DLI-X infusion to enhance graft potency and persistence, although the duration of ex vivo priming effects *in vivo* remains unclear. While the cytotoxic boost from IFN-α may improve GvL, its potential impact on GvHD risk is not fully understood and warrants further investigation. Supporting its use, a small comparative study observed that 9 out of 10 patients treated with DLI and IFN-α for relapsed CML after allo-HCT achieved complete molecular remission, compared to 3 patients who received DLI alone and experienced disease progression (133). Despite these promising findings, there have been no randomized controlled trials comparing the efficacy of DLI with and without IFN-α administration.

Short-term (e.g. 12-16h) cytokine stimulation is likely to be more beneficial and significantly more practical for DLI-XS compared to long-term (e.g. 2–4 weeks) stimulation. In our previous study on cytokine-induced killer (CIK) cells generated from resting and exercise-mobilized lymphocytes, we found no exercise effects on the expanded cell products after 21 days of culture with IL-2, following initial stimulation with IFN-γ and OKT3 (134). In that case, the intensity of cytokine stimulation and the resulting large-scale effector cell expansion likely masked any potential advantages conferred by exercise-mobilized lymphocytes. However, as methods for expanding allogeneic CIK cells continue to evolve (135), it remains plausible that exercise-mobilized lymphocytes could yield superior CIK products under alternative, less exhaustive expansion conditions. Importantly, while the concept of using cytokine stimulation to further enhance the efficacy of exercise-mobilized lymphocytes as a DLI-XS product is translationally attractive, care must be taken to avoid overactivation of T cells, which could increase the risk of GvHD and compromise safety—especially in the context of mismatched alloHCT where the therapeutic window for a beneficial GvL response is narrower.

## 8 Can DLI-X and DLI-XS synergize with other post alloHCT therapeutics?

There is a growing interest in combining DLI with other post-transplant therapies to amplify treatment effectiveness (1, 136–138). Monoclonal antibodies (mAbs), bispecific T-cell engagers (BiTEs), DNA methyltransferase inhibitors (e.g. azacitidine), tyrosine kinase inhibitors, FLT-3 inhibitors and fusion proteins are frequently employed in the treatment of myeloid and/or lymphoid malignancies. Additionally, Bcl-2 inhibitors are increasingly utilized to disrupt the anti-apoptotic defenses of tumors, rendering them more susceptible to immunotherapies. The mechanisms underlying the action of mAbs encompass several facets. These include the activation of Fcγ-receptors on innate immune cells, which leads to the opsonization of target cells, inducing lysis through cell-mediated cytotoxicity or phagocytosis. Additionally, mAbs can trigger the classical complement pathway (139). For instance, in the case of the BiTE Blinatumomab, actions involve the simultaneous binding of CD19 on cancer cells with CD3 on T-cells, facilitating the formation of an immune synapse and activating perforin-mediated T-cell cytotoxicity (TCC) (140). Other functionalities include the blocking of immune checkpoints to bolster TCC and the direct delivery of cytotoxic agents through the internalization of mAbs by target cells. However, limitations of mAbs often arise due to evident immune exhaustion, persistent MRD+, and, given their reliance on effector lymphocyte binding, inadequate cytotoxic lymphocyte frequency (141).

The enrichment of specific gene sets in DLI-X, such as those related to antigen binding and processing, T-cell receptor signaling, and response to IFN-γ (62), suggests a potential for synergy with various combination therapies aimed at enhancing the cytotoxicity of T and NK cells. Indeed, enrichment of such pathways have been associated with increased effectiveness of mAb therapies, including Blinatumomab and Rituximab, underscoring the potential for exercise to boost this response (142–144). Moreover, acute exercise has been found to increase the expression of CD16 on NK-cells, which is crucial for ADCC (65). The combination of DLI-X, which is enriched with CD16 expressing NK-cells, with mAbs designed to increase ADCC could enhance NK cell-mediated killing of tumor cells post alloHCT. Although there are no studies investigating the effects of DLI-X in conjunction with mAb therapies, a recent study by Collier-Bain et al. demonstrated in treatment-naïve CLL patients that a single session of acute cycling exercise augmented the Rituximab-mediated NK-cell lysis of autologous isolated primary CD20+ CLL cells *ex vivo* (65). This suggests that NK-cells mobilized by exercise exhibit heightened effectiveness in inducing ADCC (65).

Relapse of hematological malignancies following alloHCT may stem partly from T-cell exhaustion and the upregulation of immune checkpoints, leading to a loss of the protective GvL effect. Immune checkpoint blockade (ICB) has emerged as a frontline therapy in various solid tumors, including malignant melanoma, non-small cell lung cancer, renal cell carcinoma, and head and neck squamous cell carcinoma. The most targeted checkpoints include CTLA-4, PD-1, and PD-L1, while the efficacy of blocking other targets such as NKG2A (particularly for enhancing NK-cell function), Tim-3, and TIGIT, among others, is currently under development. In hematological cancers, anti-PD-1 inhibitors have gained approval for treating Hodgkin’s lymphoma, albeit typically after other treatments have proven ineffective. However, a primary concern with utilizing ICBs post allo-HCT is the heightened risk of GVHD (145). Given that exercise tends to mobilize highly differentiated subsets of CD8+ T-cells and NK-cells expressing elevated levels of these immune checkpoints, and that DLI-X or DLI-XS contains fewer GvHD-promoting cells, combining DLI-X or DLI-XS with an ICB could offer advantages, but this remains to be determined. Thus, while there is not yet a defined role for the prophylactic or preemptive use of ICBs to forestall post-transplant leukemic relapse, further studies may lead to identifying patient populations suitable for safely receiving ICBs post-transplant, with appropriate dosing strategies (145).

In cases of myeloid leukemias, DLI-XS may be preferred over DLI-X, as the cytokine cocktail of IL12/15/18 activates and generates CIML NK-cells within the lymphocyte collection and augments NK-cell cytotoxicity, which are effective against AML and CML. Patients may undergo combination therapies aimed at maximizing the GvL effect and/or minimizing GvHD of the DLI-X and DLI-XS products. For instance, administration of abatacept – which targets CTLA4 to inhibit T-cell co-stimulation- can be considered to mitigate GvHD and further enhance NK-cell function (146, 147). In the context of myeloid leukemias such as AML, monoclonal antibodies like lintuzumab (Anti-CD33) may be co-administered with DLI-X or DLI-XS to potentiate the ADCC effects of NK-cells. For lymphoid leukemias, monoclonal antibodies such as Tafasitamab (anti-CD19) or BiTEs like blinatumomab (anti-CD19/CD3) may enhance the efficacy of DLI-X against CD19+ B-cell malignancies including ALL or mantle cell lymphoma (148–150). It will also be important to determine the effects of using DLI-X and DLI-XS in combination with other emerging therapies to bolster the GvL effects of DLI, including DNA hypomethylation agents, Bcl-2 inhibitors, FLT-3 inhibitors, and antibody-drug conjugates, as well as those designed to inhibit GvHD such as calcineurin inhibitors, JAK and BTK inhibitors, and histone deacetylase (HDAC) inhibitors (145).

## 9 Should physical fitness be considered when selecting donors for alloHCT and cell therapy?

Donor characteristics (e.g. age, sex) are known to play an important role in patient outcomes after alloHCT (151–153). For instance, patients are less likely to relapse and have significantly less GvHD if they receive transplants from younger male donors (151, 152). While alloHCT donors are chosen based on HLA matching, emphasizing their overall health is crucial for successful stem cell mobilization and collection procedures. Instances where multiple donors are available, such as haploHCT involving either parent or a sibling, pose a decision-making challenge for physicians in determining the most suitable donor. The influence of donor physical fitness, particularly metrics such as V̇O_2_max, on patient outcomes following alloHCT remains largely unexplored. Considering the association between V̇O_2_max and enhancements in various immunological functions, such as NK-cell cytotoxicity, T-cell polyfunctionality, and proliferation, it is reasonable to anticipate improved patient outcomes (e.g., reduced GvHD, lower relapse rates, and earlier immune reconstitution) in patients whose donors are more physically fit (6). Since V̇O_2_max, a modifiable factor that is associated with lower all-cause mortality and correlates with enhanced T-cell and NK cell function throughout life (6, 154), we suggest that donor V̇O_2_max could become a key independent predictor of patient outcomes after alloHCT. While most studies to date that have attempted to collect and/or manufacture therapeutic cell products from exercising donors have used relatively fit, healthy individuals, a recent study found that physical activity status had a major impact on the *ex vivo* expansion of γδ T-cells for allogeneic cell therapy (155). Specifically, γδ T-cell expansions were successful in all participants performing physical activity >4days/week, while successful expansions occurred in only 25% of donors performing physical activity <4 days/week (155). Given that acute exercise mobilization enhances the *ex vivo* expansion of γδ T-cells and other therapeutic cell products (e.g., DLI-X) in physically active individuals (62, 128, 156), this short-term intervention may actually be more effective in individuals with lower physical fitness and/or activity levels; however, this requires further investigation.

While other fitness components (e.g., muscular strength, muscular endurance, and body composition) also influence immune function and may affect donor suitability, V̇O_2_max stands out due to its strong association with overall health and reduced all-cause mortality. Additionally, cardiorespiratory-based exercise is known to elicit more pronounced changes in immune function compared to other forms of exercise, making it a compelling focus for research aimed at improving alloHCT outcomes (110). If, as expected, V̇O_2_max is identified as an independent predictor of DLI safety and efficacy, alloHCT donors could benefit from targeted exercise training prescriptions to enhance their V̇O_2_max before donating cells for therapy. Considering that DLIs are typically performed around 90 days post-alloHCT, this timeframe would allow for the implementation of an effective training program tailored to the alloHCT donor population.

## 10 Conclusion

**Figure 2:**
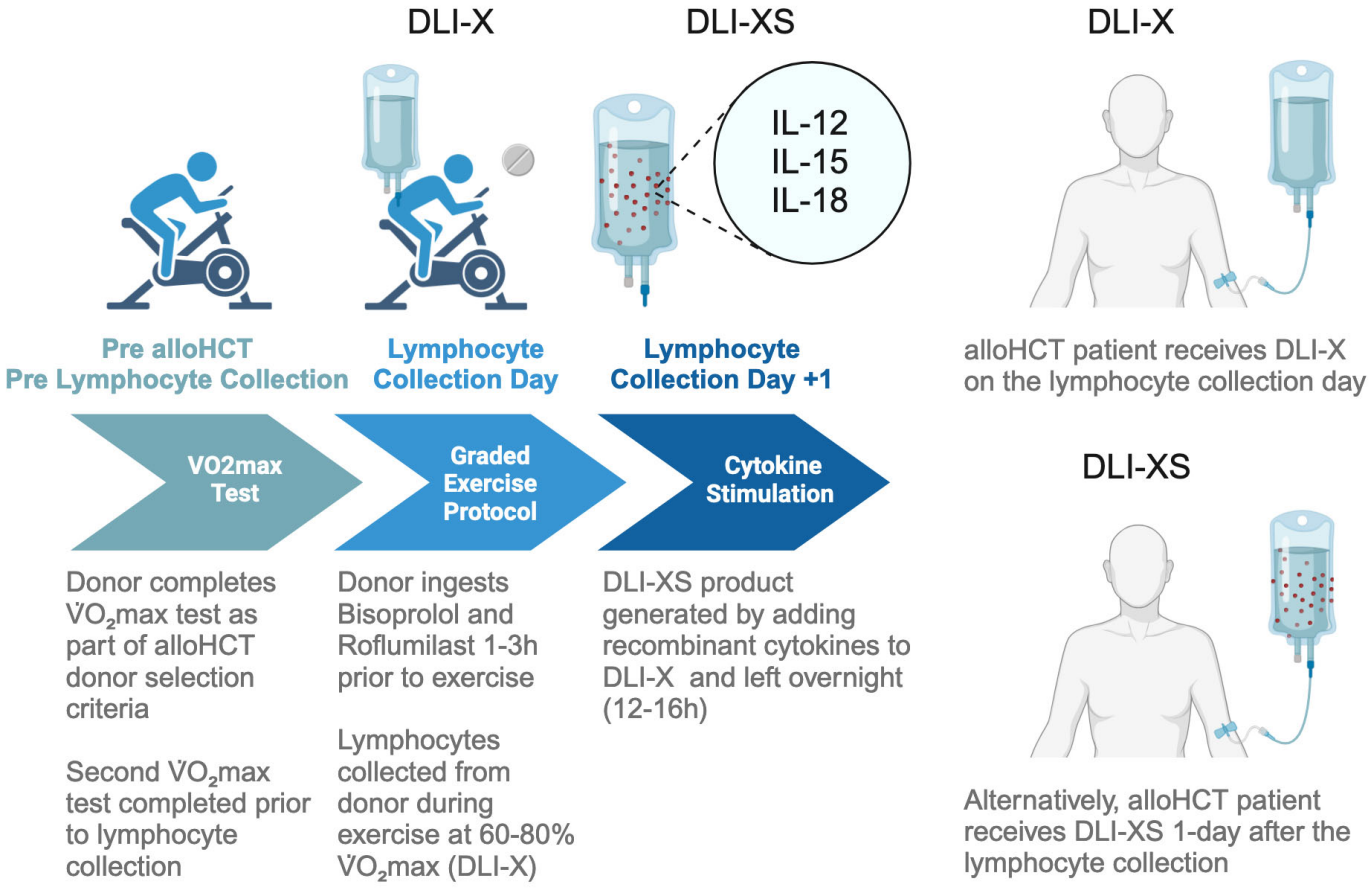
Workflow for collecting lymphocytes from exercising donors for DLI-X and DLI-XS applications. The process begins with HLA typing and cardiorespiratory fitness assessment of potential allo-HCT donors using a graded exercise test. The donor selected for HCT is a matched sibling or haploidentical donor with optimal characteristics, including a high HLA match, younger age, male gender, and superior cardiorespiratory fitness. When prescribing exercise intensities for the lymphocyte collection procedures, a secondary V̇O_2_max test may be performed closer to the lymphocyte collection day if the donor’s activity level has significantly changed. On collection day, donors receive 10 mg of bisoprolol and 250 mcg of roflumilast at three and two hours prior to exercise, respectively, timed to achieve peak plasma drug concentrations (157, 158). Bisoprolol enhances epinephrine availability via β1-AR blockade, favoring β2-AR signaling and increasing the mobilization of NK cells, which raises the NK/T-cell ratio in DLI-X. Roflumilast amplifies cAMP-PKA signaling, enhancing γδ T-cell mobilization and lowering the CD4+/CD8+ T-cell ratio. Donors complete a graded cycling ergometer protocol (50–80% V̇O_2_max) as detailed in the ‘Allo-X’ trial (NCT06643221). Blood is collected via a peripheral vein using a whole blood collection set into anticoagulant-treated bags. While leukapheresis is traditionally used for DLI lymphocyte collection, a recent study has highlighted the safety and efficacy of collecting whole blood units from haploidentical donors (159). For DLI-X, freshly collected lymphocytes are directly infused into recipients. Donors may perform repeat exercise bouts to provide additional units if needed. For DLI-XS, lymphocytes are stimulated overnight with IL-12, IL-15, and IL-18 before infusion. Patients may receive DLI-X or DLI-XS doses prophylactically, preemptively, or therapeutically based on clinical requirements.

For more than three decades, DLI has stood as a cornerstone in post-transplant therapeutic strategies for addressing leukemic relapse. The advantages of DLI are manifold: readily availability of donors in the matched related setting, its capacity to stimulate GvL effects resulting in durable remissions, and the potential for achieving a cure. Nonetheless, DLI also presents notable drawbacks, including the substantial risk of GvHD, its limited efficacy—especially evident in cases of refractory leukemia—and the delay in therapeutic response. In this review, we have proposed a novel approach to address these challenges by harnessing lymphocytes collected from healthy donors during a single bout of moderate to vigorous intensity exercise (DLI-X). This strategy offers simplicity and cost-effectiveness. Additionally, by enhancing β2-AR signaling during exercise with a selective β1-AR antagonist and a PDE4 inhibitor, and by augmenting the potency of collected lymphocytes through *ex vivo* stimulation with NK-cell enhancing cytokines (DLI-XS), we anticipate even better clinical outcomes for patients (**Figure 2**). While much of the data presented here is derived from our group’s work, it is important to note that the exploration of DLI-X and DLI-XS represents an evolving field, and further studies from other research groups will be crucial in validating and refining these strategies. Looking ahead, it is imperative to assess the effectiveness of DLI-X and DLI-XS in a clinical trial to determine whether lymphocytes mobilized during exercise can genuinely prevent and treat leukemic relapse post-alloHCT to a greater extent than standard DLI.

## Data Availability

The raw data supporting the conclusions of this article will be made available by the authors, without undue reservation.
